# Quality and reliability of pancreaticoduodenectomy-related videos on TikTok, Bilibili, and YouTube: a cross-sectional content analysis

**DOI:** 10.3389/fsurg.2026.1898401

**Published:** 2026-09-09

**Authors:** Shuo Yang, Xiong Teng, Rui Yang, Jiayi Chen, Shihao Jiang, Songling Liu, Chao Chen, Shengyu Liu, Yi Xiao, Wei Cheng, Lei Zhou

**Affiliations:** 1 Department of Hepatobiliary Surgery, Hunan Provincial People’s Hospital, The First Affiliated Hospital of Hunan Normal University, Changsha, China; 2 School of Medicine, Shanxi Datong University, Datong, China

**Keywords:** health information, information quality, medical misinformation, pancreaticoduodenectomy, patient education, short video, social media, Whipple procedure

## Abstract

**Background:**

Pancreaticoduodenectomy (PD), also known as the Whipple procedure, is a complex surgical procedure for pancreatic head and periampullary lesions. As short-video platforms increasingly shape the way patients and the public access health information, the quality and reliability of PD-related videos have important implications for patient education and public health communication.

**Objective:**

This study aimed to evaluate the source, content coverage, quality, reliability, and engagement characteristics of PD-related short videos on TikTok, Bilibili, and YouTube.

**Methods:**

Searches were conducted from January 26 to March 16, 2026, using newly registered accounts and Chinese and English keywords related to PD. After predefined inclusion and exclusion criteria were applied, 180 videos were included. Two independent raters with medical backgrounds assessed content coverage, the Global Quality Score (GQS), and the modified DISCERN (mDISCERN) score. Final GQS and mDISCERN scores were calculated as the average of the two raters’ scores. Engagement indicators and uploader characteristics were extracted. Mann–Whitney U tests, Kruskal–Wallis H tests, chi-square tests, Spearman correlation analyses, interrater reliability analyses, and exploratory ROC and multivariable regression analyses were performed.

**Results:**

Interrater agreement was excellent for both GQS and mDISCERN. Among the 180 videos, 159 (88.33%) were uploaded by medical professionals and 21 (11.67%) by non-medical professionals. Medical-professional videos had significantly more likes and comments. In unadjusted comparisons, non-medical videos were longer and had higher GQS scores; however, uploader type was not independently associated with GQS after adjustment for duration and platform. mDISCERN scores did not differ significantly between the two groups. Most videos focused on management or procedural aspects of PD, whereas definitions, symptoms/indications, and outcomes/prognosis were less frequently addressed. Video duration was positively correlated with GQS and mDISCERN, and a sample-derived threshold of 160 s identified videos with GQS ≥4 with an AUC of 0.886. Significant differences in duration, engagement, quality scores, and uploader distribution were observed across platforms.

**Conclusion:**

PD-related videos on TikTok, Bilibili, and YouTube have some educational value but remain limited by incomplete content coverage and inconsistent reliability. Improving the completeness, transparency, comprehensibility, and platform governance of surgical health videos may help patients access more trustworthy information.

## 1 Introduction

Pancreatic cancer is a highly malignant gastrointestinal tumor characterized by an insidious onset, rapid progression, increasing global disease burden, and poor prognosis (1). For patients with pancreatic head and periampullary lesions, pancreaticoduodenectomy (PD) remains one of the most important surgical treatments with curative potential (2–4). However, PD is technically demanding and associated with substantial perioperative risk, including postoperative hemorrhage, pancreatic fistula, infection, delayed gastric emptying, and nutritional challenges (3–5). Patients and their families therefore require reliable information about the procedure, indications, perioperative risks, postoperative recovery, and long-term outcomes.

Traditional medical communication relies largely on outpatient counseling, written educational materials, and face-to-face explanation. Because surgical terminology and anatomical concepts are often difficult for patients to understand, digital media and short-video platforms have become increasingly important sources of health information. Patients and the general public frequently use digital platforms to supplement clinical consultations, which may influence disease perception, physician-patient communication, and treatment-related decision-making (6–8). Compared with text-based information, videos can present surgical procedures, anatomy, and perioperative precautions in a more intuitive format and may therefore have educational value in complex surgical topics.

Nevertheless, health information on social media is not always accurate, complete, or evidence-based. Prior research has shown that social media can amplify medical misinformation and incomplete health information, potentially affecting users’ understanding of diseases and treatments (9, 10). Studies of TikTok, Bilibili, and YouTube have also demonstrated that health-related videos are highly accessible but vary substantially in quality, completeness, and reliability (11–13). Evaluating PD-related videos across major video platforms is therefore relevant not only to surgical education but also to broader public health communication and digital health governance. This cross-sectional content analysis assessed the source, content coverage, quality, reliability, and engagement characteristics of PD-related videos on TikTok, Bilibili, and YouTube.

## 2 Materials and methods

### 2.1 Search strategy and data collection

Videos were searched on TikTok, Bilibili, and YouTube using Chinese and English keywords related to PD, including “pancreaticoduodenectomy,” “Whipple procedure,” “Whipple surgery,” “胰十二指肠切除术,” “胰头十二指肠切除术,” and “惠普尔手术.” Searches were conducted from January 26 to March 16, 2026. To reduce the potential influence of browsing history and personalization algorithms, newly registered accounts were used on all platforms, and default platform settings and default ranking orders were retained as far as possible. The search strategy was centered on PD across all platforms to improve comparability, while allowing minor adjustments in keyword order to reflect platform-specific search mechanisms. Similar cross-sectional content analysis methods have been used in previous health information quality studies of short-video platforms (11–13). A separate literature search was conducted and updated from January 23 to May 6, 2026 to inform the study design, support the selection of assessment methods, and contextualize the findings. References retrieved after completion of video data extraction were used only to update the background and discussion and did not influence video selection, data extraction, scoring, or statistical analysis. Use of newly registered accounts reduced the influence of prior browsing history and personalization, but it could not eliminate platform-specific ranking effects. Accordingly, the first 100 eligible videos on TikTok and YouTube represented highly ranked content under the default search conditions during the study period rather than a random sample of all available videos. Search position may still have been influenced by popularity, recency, geographic location, language, and proprietary platform algorithms.

**Figure 1:**
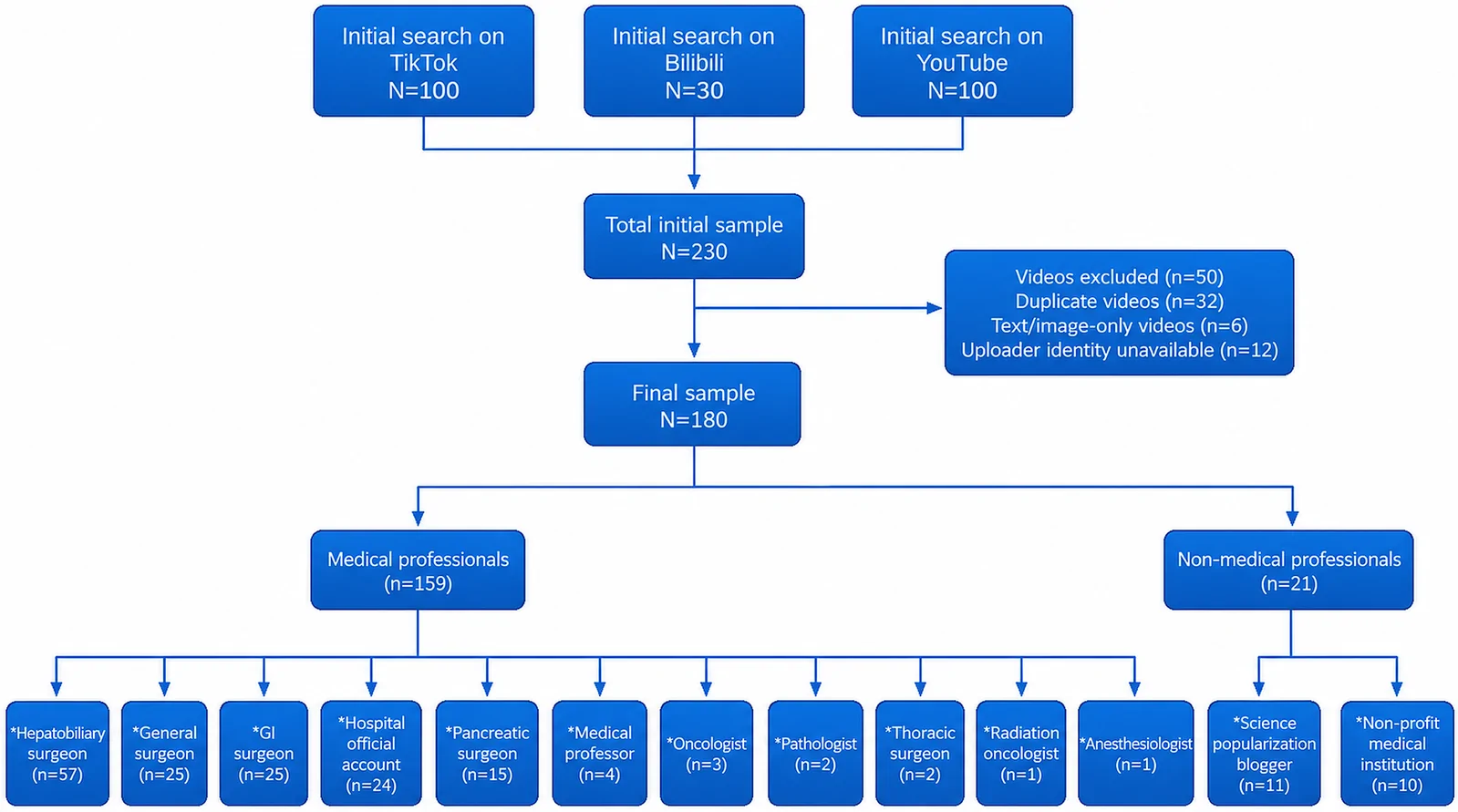
Video screening procedure. The flow diagram shows the initial retrieval, exclusion process, and final sample included from TikTok, Bilibili, and YouTube.

The initial plan was to include the first 100 relevant videos from each platform. During the actual search process, however, only 30 eligible Bilibili videos directly related to PD were identified. To prioritize topical relevance and content validity, the final sample consisted of the first 100 eligible TikTok videos, all 30 eligible Bilibili videos, and the first 100 eligible YouTube videos before duplicate and exclusion screening. The final analytical sample contained 180 videos. The video screening procedure is summarized in Figure 1.

Videos were included if they were related to PD and contained explanatory content. Videos were excluded if they were irrelevant to the study topic or lacked useful information, consisted only of images, subtitles, or screen displays without substantive explanation, were duplicates, or had uploader identity that could not be determined. Inclusion and exclusion criteria were predefined before formal screening. For each video, uploader identity, video duration, likes, comments, collections, and shares were extracted. Because some platforms did not display all engagement indicators, collections and shares were analyzed only when available. All data were entered into Microsoft Excel for organization.

### 2.2 Assessment indicators

Videos were evaluated in three dimensions: content coverage, information quality, and information reliability. Content coverage was classified into four categories according to previous short-video health information studies and the educational needs of patients undergoing PD: Definition, Symptoms/Indications, Management, and Outcomes (11–13). Definition referred to the basic concept of PD, relevant anatomy, or disease background. Symptoms/Indications included symptoms, indications, or reasons for receiving surgery. Management included surgical procedures, perioperative management, and treatment strategies. Outcomes referred to postoperative recovery, complications, and prognosis. This framework is consistent with broader dimensions of online health information quality, including reliability, sufficiency, comprehensibility, and transparency (14).

The modified DISCERN (mDISCERN) tool was used to evaluate overall reliability. DISCERN and its modified versions have been widely used to assess the reliability of consumer health information (15). The mDISCERN tool includes five items evaluating clarity, relevance, traceability of information sources, evidence support, and objectivity. Each item was scored as 1 for “yes” and 0 for “no,” yielding a total score from 0 to 5. The Global Quality Score (GQS) was used to assess overall video quality. GQS is a five-point scale, with higher scores indicating more complete, better organized, and more useful information. Both mDISCERN and GQS were independently assessed by the same two raters using predefined criteria. The final mDISCERN and GQS scores used in the analyses were calculated as the average of the two raters’ total scores. When substantial discrepancies were observed, a third researcher reviewed the ratings to identify potential scoring errors and facilitate adjudication. Prior studies of YouTube, TikTok, and short-video health information have adopted GQS, DISCERN, or related instruments to evaluate video quality and reliability (12, 13, 16, 17).

### 2.3 Rating process

Before formal evaluation, two raters (YS and YR), both with medical backgrounds, received standardized training in the mDISCERN, GQS, and content-coding criteria. To reduce the potential influence of prior exposure to PD-related platform content, the raters had not systematically browsed PD-related videos on the included platforms before the formal search and assessment. After the predefined inclusion and exclusion criteria were applied, 180 videos were included for analysis.

Videos were classified as uploaded by medical professionals or non-medical professionals according to uploader identity. Medical professionals included hepatobiliary surgeons, gastrointestinal surgeons, hospital official accounts, general surgeons, pancreatic surgeons, medical professors, oncologists, pathologists, thoracic surgeons, radiation oncologists, and anesthesiologists. Non-medical uploaders included science popularization bloggers and non-profit medical institutions. Descriptive analyses retained detailed uploader categories to reflect the actual distribution of video sources. For inferential analyses, medical-professional subgroups with very small sample sizes were combined into an “other medical professionals” category to improve the stability of between-group comparisons.

The two raters independently recorded basic video information and assessed content coverage, GQS, and mDISCERN scores. The final GQS and mDISCERN scores for statistical analyses were calculated as the average of the two raters’ scores; substantial discrepancies were reviewed by a third researcher to identify scoring errors and facilitate adjudication.

### 2.4 Statistical analysis

**Figure 2:**
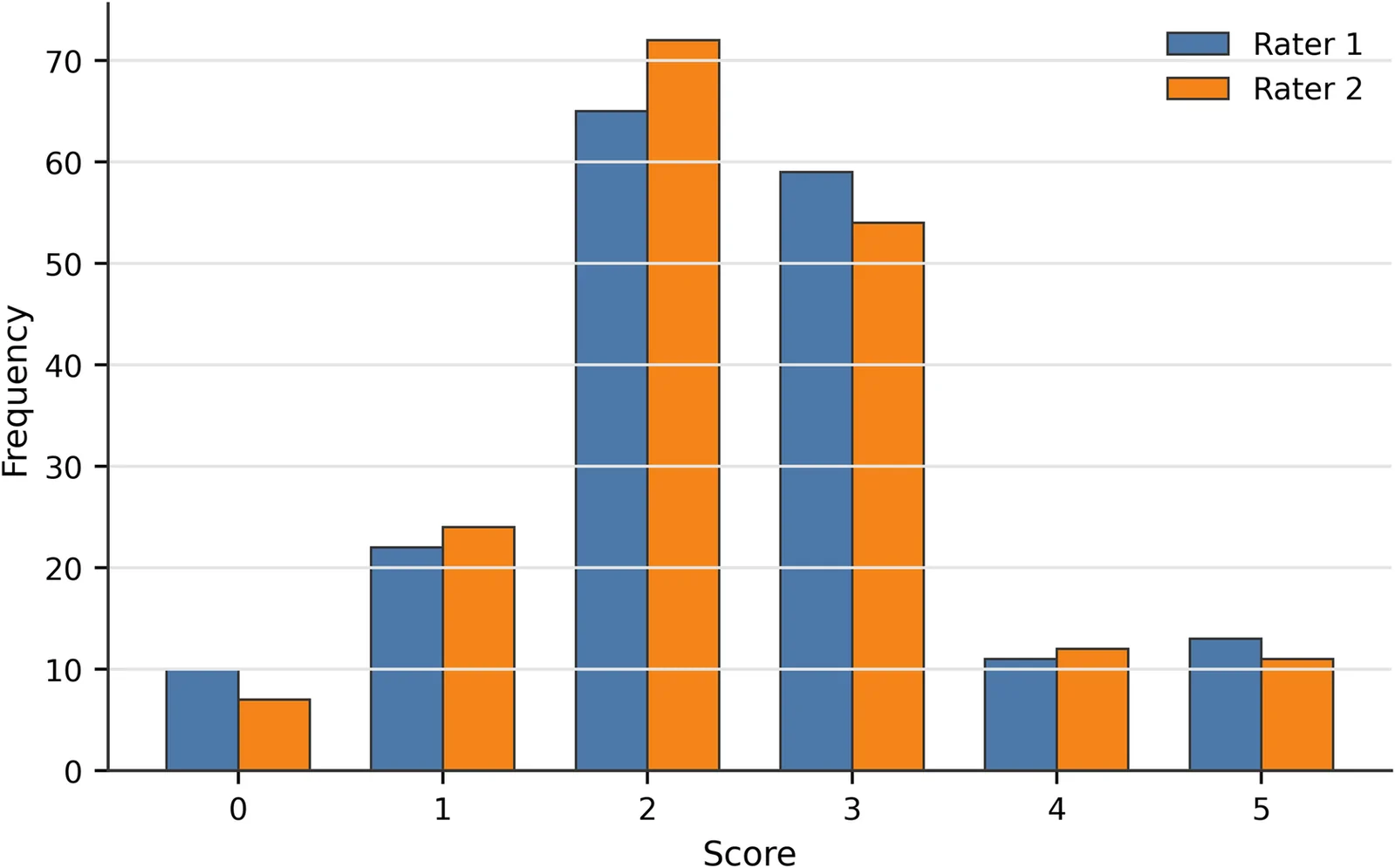
Comparison of mDISCERN score distributions between two raters. The figure shows the distribution of total mDISCERN scores assigned by the two independent raters across different score categories.

Interrater agreement for GQS and mDISCERN total scores was assessed using the single-measure intraclass correlation coefficient (ICC) based on a two-way random-effects model for absolute agreement. Agreement was excellent for both GQS (ICC = 0.905) and mDISCERN (ICC = 0.939). Statistical analyses were performed using SPSS 24.0. Categorical variables were expressed as *n* (%), and non-normally distributed continuous variables were expressed as medians and interquartile ranges. Continuous variables were compared between medical and non-medical uploader groups using Mann–Whitney U tests. Continuous variables across the three platforms were compared using Kruskal–Wallis H tests. When overall platform differences were significant, exploratory *post-hoc* pairwise comparisons were performed using Dunn tests with Bonferroni correction. Differences in uploader type distribution across platforms were assessed using chi-square tests, and exploratory pairwise comparisons were performed using Fisher's exact tests with Bonferroni correction. Spearman correlation analysis was used to evaluate relationships between video duration and engagement indicators, GQS, and mDISCERN. A two-sided *p* value <0.05 was considered statistically significant. Because multiple platform comparisons were exploratory, adjusted *p* values were reported and interpreted cautiously. The mDISCERN score distributions assigned by the two independent raters are shown in Figure 2. Two additional *post hoc* exploratory analyses were conducted. First, receiver operating characteristic (ROC) analysis evaluated video duration as a discriminator of high-quality content, operationalized as a final GQS of 4 or higher (good-to-excellent quality). The duration threshold that maximized the Youden index was reported together with the area under the curve (AUC), a 95% bootstrap confidence interval based on 5,000 resamples, sensitivity, and specificity. Second, a multivariable linear regression sensitivity analysis with heteroscedasticity-robust (HC3) standard errors assessed whether uploader type remained associated with final GQS after adjustment for log-transformed video duration [ln(duration+1)] and platform. The primary analyses were performed using SPSS 24.0, and the *post hoc* analyses were performed using Python 3.13.5 with scikit-learn 1.8.0 and statsmodels 0.14.6. These *post hoc* analyses were considered exploratory.

## 3 Results

### 3.1 Video sources and general characteristics

A total of 180 PD-related videos were included. Of these, 159 (88.33%) were uploaded by medical professionals and 21 (11.67%) by non-medical professionals. Among medical professionals, hepatobiliary surgeons were the most common uploaders (57 videos, 31.77%), followed by general surgeons (25 videos, 13.89%), gastrointestinal surgeons (25 videos, 13.89%), hospital official accounts (24 videos, 13.33%), pancreatic surgeons (15 videos, 8.33%), medical professors (4 videos, 2.22%), oncologists (3 videos, 1.67%), pathologists (2 videos, 1.11%), thoracic surgeons (2 videos, 1.11%), radiation oncologists (1 video, 0.56%), and anesthesiologists (1 video, 0.56%). Among non-medical uploaders, 11 videos (6.11%) were uploaded by science popularization bloggers and 10 (5.56%) by non-profit medical institutions. Detailed characteristics by uploader source are presented in Table 1.

**Table 1:** Basic characteristics of videos by uploader source.

Source	*n* (%)	Duration (s) Median (IQR)	Likes Median (IQR)	Collections Median (IQR)	Shares Median (IQR)	Comments Median (IQR)
HPB surgeon	57 (31.77)	64 (37–103)	168 (63–988)	88 (14.5–564)	49 (8–515)	20 (7–252)
General surgeon	25 (13.89)	82 (49–222)	227 (45–377)	66 (8–152)	32.5 (3–169)	8 (2–40)
GI surgeon	25 (13.89)	20 (5–76.75)	76 (40–185)	20 (5–76.75)	12.5 (4–87.25)	8 (4–21)
Hospital account	24 (13.33)	228 (75.75–544.25)	50.5 (7–399.75)	3 (1.5–315.5)	635 (318–1809.5)	0 (0–6)
Pancreatic surgeon	15 (8.33)	99 (69–239.5)	300 (216–709)	100 (61.25–483.75)	78 (36.5–391)	46 (14–127.5)
Medical professor	4 (2.22)	925 (506–1466.75)	923 (149.75–2505.5)	1191 (1191-1191)	602 (602-602)	63 (9–333.75)
Oncol.	3 (1.67)	61 (55–215)	11 (6.5–62)	31 (31-31)	36 (36-36)	1 (0.5–4.5)
Pathol.	2 (1.11)	132.5 (122.25–142.75)	373 (190–556)	41.5 (21.75–61.25)	32.5 (16.75–48.25)	42.5 (21.75–63.25)
Thoracic surgeon	2 (1.11)	86.5 (86.25–86.75)	3554.5 (3473.25–3635.75)	1793.5 (1641.75–1945.25)	1431.5 (1316.75–1546.25)	287.5 (197.25–377.75)
Rad. oncol.	1 (0.56)	238 (238-238)	1633 (1633-1633)	257 (257–257)	112 (112-112)	852 (852–852)
Anesth.	1 (0.56)	733 (733-733)	5 (5-5)	11 (11–11)	2 (2-2)	1 (1–1)
Science blogger	11 (6.11)	299 (82–525.5)	32 (19–142)	41 (36–162)	31 (5–92)	3 (1–8)
Non-profit institution	10 (5.56)	217.5 (172–418.5)	36 (1–401.75)	NA	NA	0 (0–9.5)

Some platforms did not display all engagement indicators; therefore, collections or shares were unavailable for some uploader sources. This table presents descriptive statistics and retains detailed uploader categories. In inferential between-group analyses, medical-professional subgroups with small sample sizes were combined into the “other medical professionals” category. HPB, hepatobiliary; GI, gastrointestinal; Oncol., oncologist; Pathol., pathologist; Rad., radiation; Anesth., anesthesiologist.

Uploader subgroups were unevenly distributed. Hepatobiliary surgeons, general surgeons, gastrointestinal surgeons, hospital official accounts, and pancreatic surgeons constituted the main video sources, whereas thoracic surgeons, pathologists, radiation oncologists, and anesthesiologists contributed relatively few videos. Detailed subgroup distributions were therefore retained for descriptive analysis, while small medical-professional subgroups were combined for inferential comparisons.

**Figure 3:**
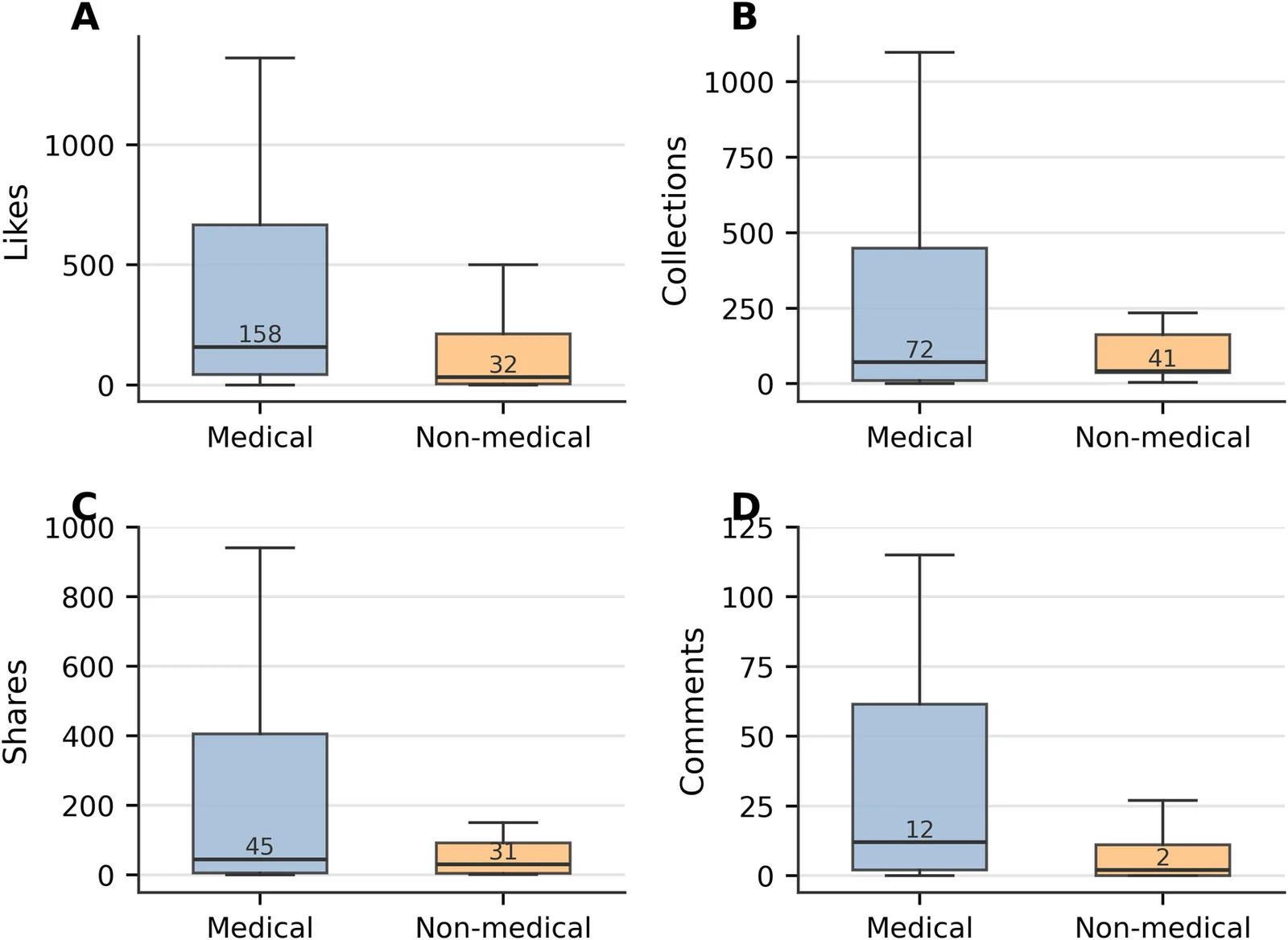
Comparison of engagement indicators between videos uploaded by medical and non-medical professionals: **(A)** likes, **(B)** collections, **(C)** shares, and **(D)** comments. Median values with interquartile ranges are shown because these engagement indicators were non-normally distributed; inferential comparisons were based on Mann-Whitney U tests. Collections and shares were calculated only for videos with available data.

Across all included videos, there were 159,675 likes and 22,914 comments. Among the 117 videos with available collection and share data, there were 43,130 collections and 44,757 shares. Videos uploaded by medical professionals had significantly more likes than those uploaded by non-medical professionals [158 (44–666) vs. 32 (5–213), U = 2308.5, *p* = 0.004] and significantly more comments [12 (2–61.5) vs. 2 (0–11), U = 2399.0, *p* = 0.001]. Collections [72 (10.75–448.25) vs. 41 (36–162), U = 519.0, *p* = 0.740] and shares [45 (5.75–405.25) vs. 31 (5–92), U = 580.0, *p* = 0.339] did not differ significantly between groups. Videos uploaded by non-medical professionals were significantly longer than those uploaded by medical professionals [226 (106–484) s vs. 82 (50.5–239.5) s, U = 1121.0, *p* = 0.015]. Engagement indicators by uploader type are shown in Figure 3.

Spearman correlation analysis showed that video duration was not significantly correlated with likes (r = 0.053, *p* = 0.476) or shares (r = 0.145, *p* = 0.118). However, video duration was weakly but significantly correlated with collections (r = 0.251, *p* = 0.006), suggesting that longer videos were more likely to be saved by users.

### 3.2 Content coverage

Because individual videos could cover multiple content categories, the sum across content dimensions could exceed the number of videos in a given source category. Overall, video content was concentrated in the Management dimension, including surgical procedures, treatment methods, and perioperative management, whereas Definition, Symptoms/Indications, and Outcomes were less frequently covered.

Because medical-professional videos accounted for most of the sample, they contributed the largest absolute number of content items. However, when considered relative to group size, non-medical videos showed comparable content coverage, particularly in Symptoms/Indications, Management, and Outcomes. Therefore, differences in content coverage should be interpreted descriptively. In particular, videos uploaded by hepatobiliary surgeons, hospital official accounts, and pancreatic surgeons provided relatively greater absolute coverage of Management and Outcomes, while hospital official account videos involved multiple dimensions and showed relatively greater systematicity. Detailed content coverage by uploader source is summarized in Table 2.

**Table 2:** Content coverage of videos by uploader source.

Uploader source	Definition	Symptoms/Indications	Management	Outcomes
Hepatobiliary surgeon (*n* = 57)	13	22	34	20
General surgeon (*n* = 25)	4	6	19	7
Gastrointestinal surgeon (*n* = 25)	5	5	19	7
Hospital official account (*n* = 24)	13	10	21	11
Pancreatic surgeon (*n* = 15)	3	4	12	7
Medical professor (*n* = 4)	4	2	4	2
Oncologist (*n* = 3)	1	0	3	1
Pathologist (*n* = 2)	0	0	0	2
Thoracic surgeon (*n* = 2)	0	0	2	0
Radiation oncologist (*n* = 1)	1	1	1	1
Anesthesiologist (*n* = 1)	1	0	1	1
Science popularization blogger (*n* = 11)	2	5	8	7
Non-profit medical institution (*n* = 10)	1	4	9	5

A single video could cover more than one content dimension; therefore, the sum across columns may exceed the total number of videos from a given source. This table presents descriptive statistics and retains detailed uploader categories. In inferential between-group analyses, medical-professional subgroups with small sample sizes were combined into the “other medical professionals” category.

### 3.3 Video quality and reliability

The mean GQS score, calculated as the average of the two raters’ scores, was 2.72 for videos uploaded by medical professionals and 3.24 for those uploaded by non-medical professionals. Descriptively, videos uploaded by medical professionals were mainly concentrated at GQS scores of 2 to 3.5, whereas non-medical videos were more frequently concentrated at scores of 2.5 to 4. Mann–Whitney U testing showed that GQS scores were significantly higher among non-medical videos than among medical professional videos [3 (2.5–4) vs. 2.5 (2–3.5), U = 1198.5, *p* = 0.033].

The mean mDISCERN scores, calculated as the average of the two raters’ total scores, were 2.41 for medical professional videos and 2.52 for non-medical videos. The difference was not statistically significant [2 (2–3) vs. 3 (2–3), U = 1490.5, *p* = 0.412], indicating that the reliability of videos from both uploader groups remained generally moderate to low.

**Figure 4:**
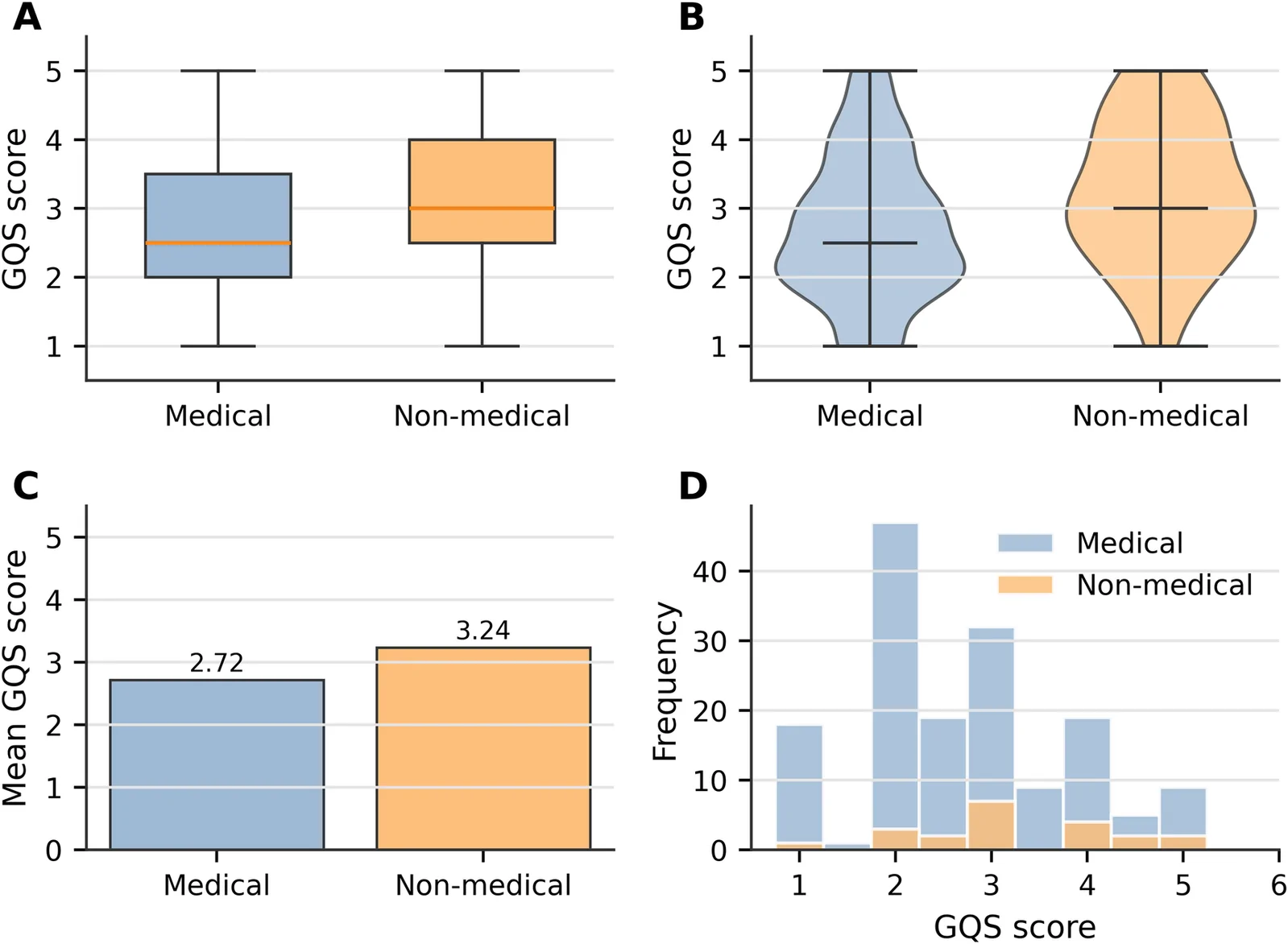
Distribution of GQS scores by uploader type: **(A)** boxplot, **(B)** violin plot, **(C)** mean GQS score bar chart, and **(D)** GQS score frequency distribution. These panels summarize differences in final GQS scores between medical and non-medical uploaders. Final GQS scores were calculated as the average of the two raters' scores.

**Figure 5:**
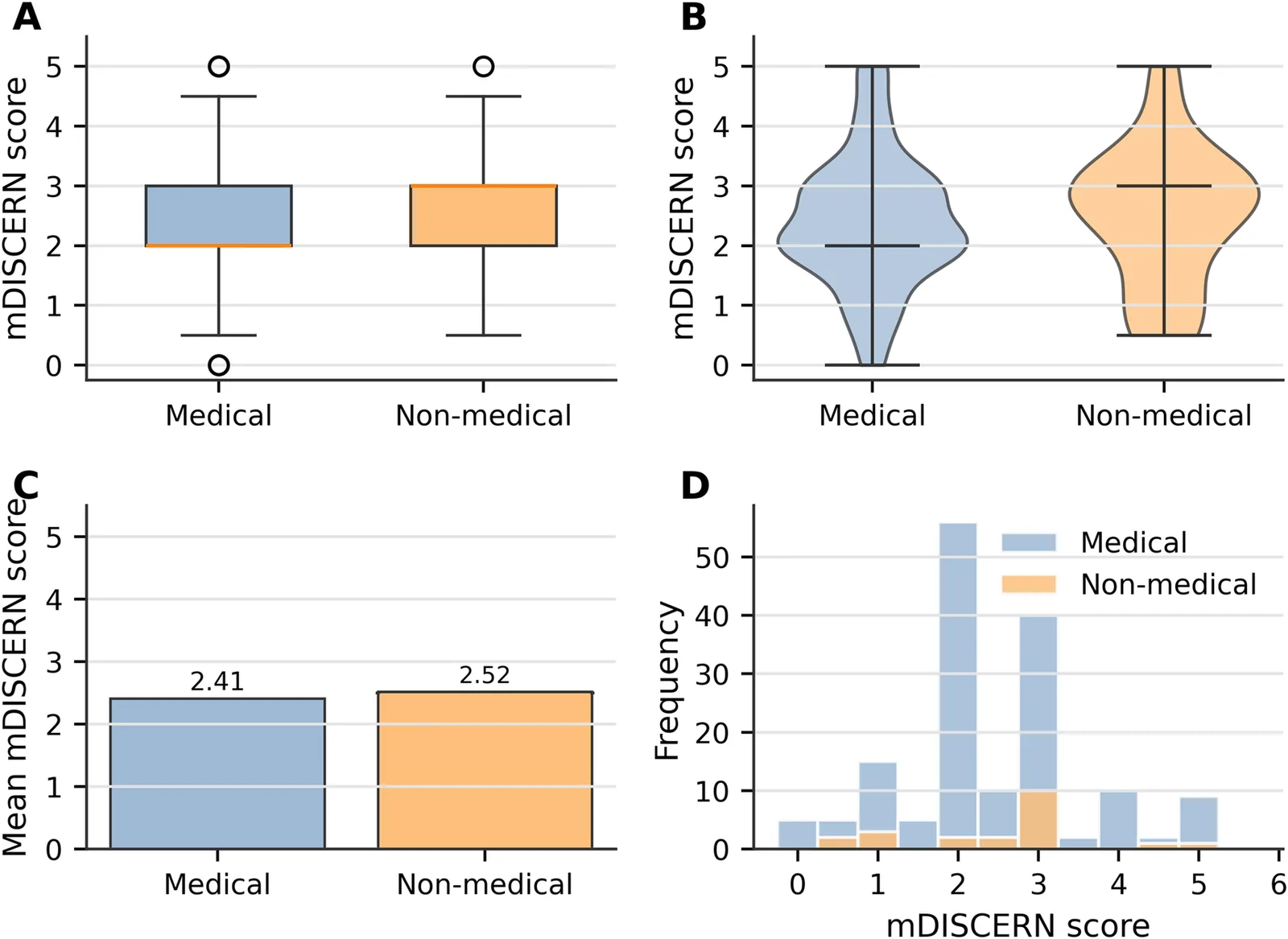
Distribution of final mDISCERN scores by uploader type. Boxplot, violin plot, bar chart, and frequency distribution are used to summarize differences in final mDISCERN scores between medical and non-medical uploaders. Final mDISCERN scores were calculated as the average of the two raters’ total scores.

**Figure 6:**
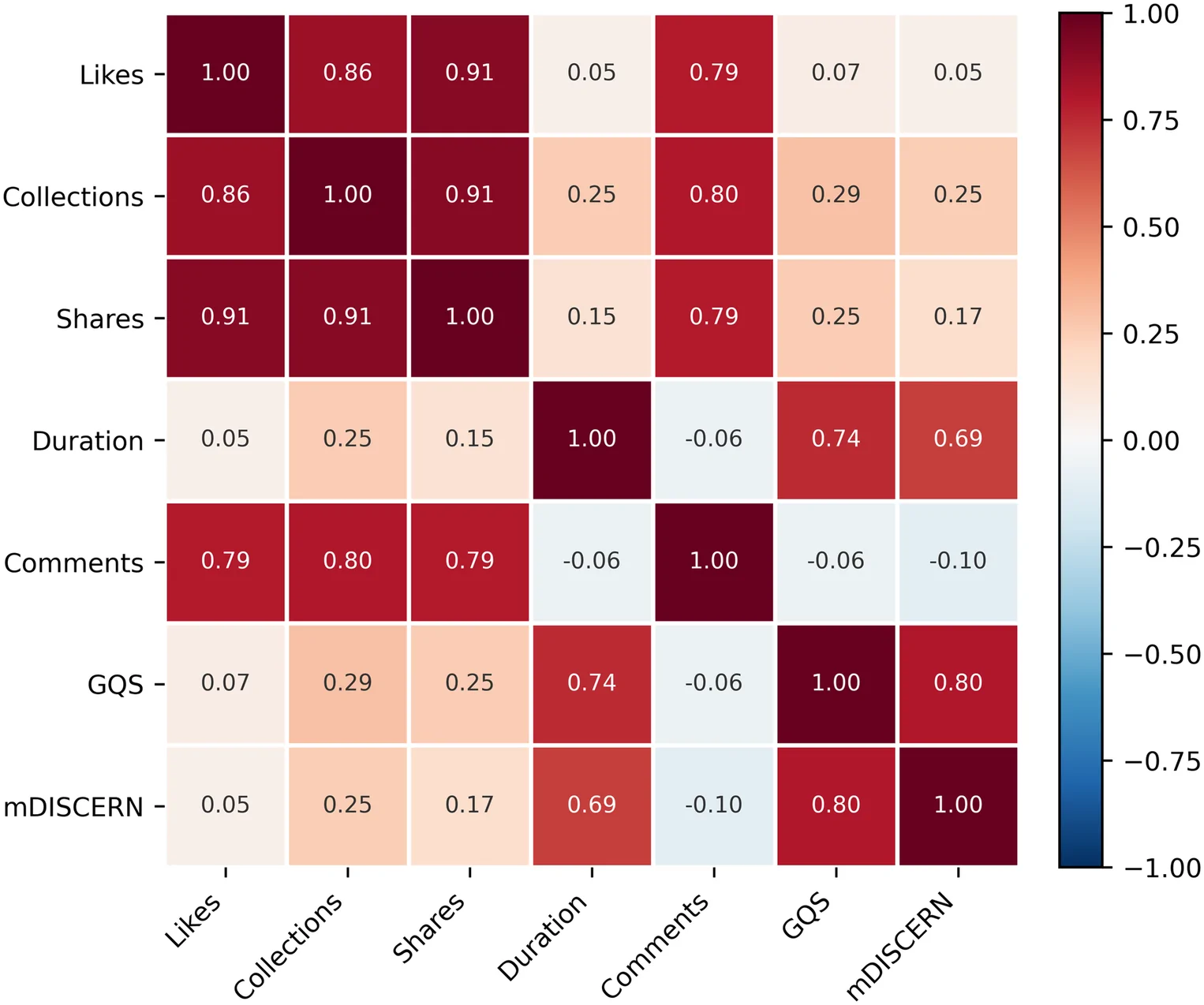
Spearman correlation heatmap of engagement indicators, video duration, and quality scores. The heatmap summarizes pairwise Spearman correlations among likes, collections, shares, comments, video duration, final GQS score, and final mDISCERN score. Pairwise complete observations were used because collections and shares were unavailable for some videos.

**Figure 7:**
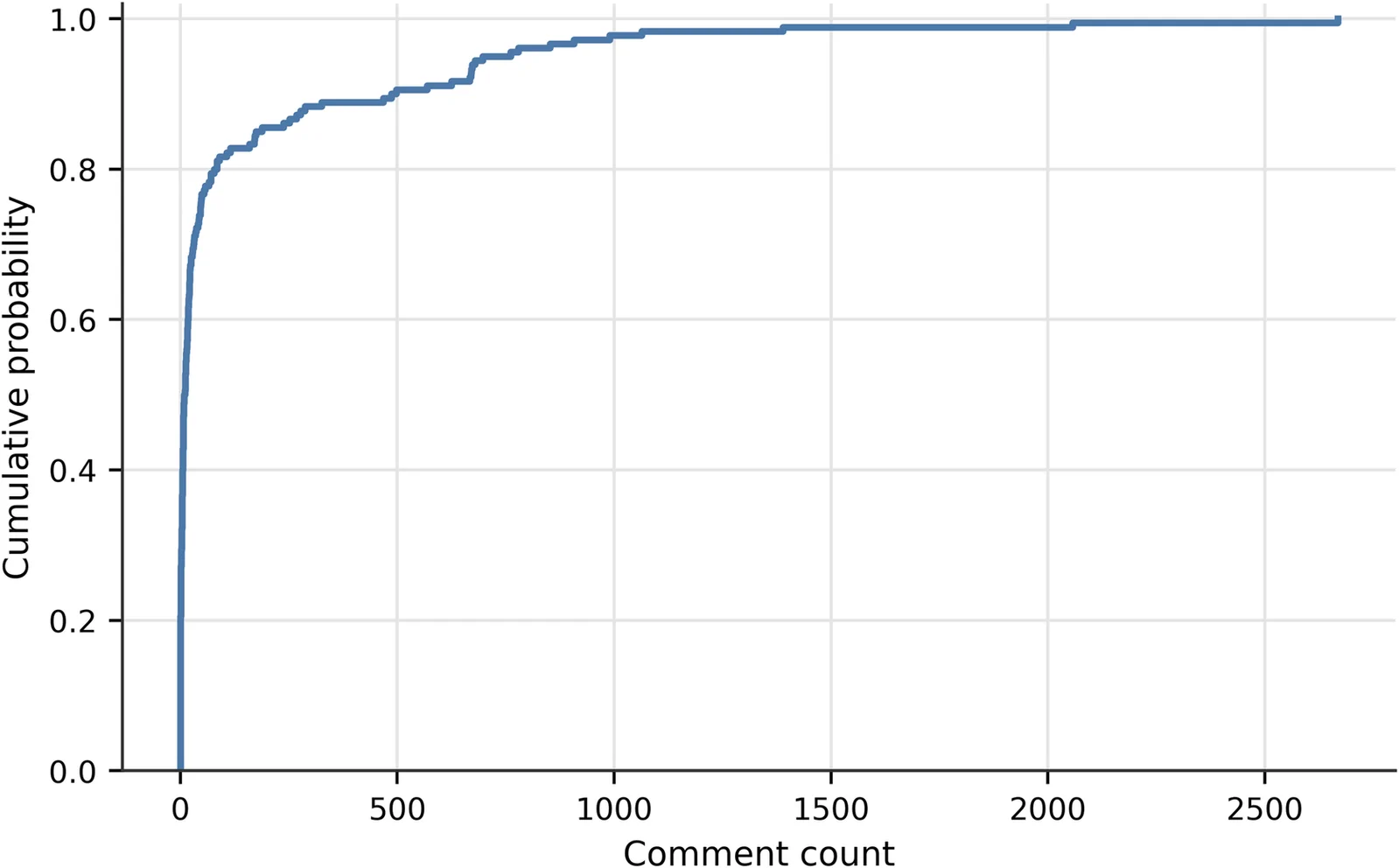
Cumulative distribution of comment counts. The figure shows the cumulative distribution pattern of comment counts across included videos.

GQS and mDISCERN scores were strongly correlated in videos uploaded by medical professionals (r = 0.8060, *p* < 0.001) and strongly correlated in videos uploaded by non-medical professionals (r = 0.7195, *p* < 0.001). Video duration was strongly correlated with GQS (rho=0.7434, *p* < 0.001) and moderately to strongly correlated with mDISCERN (rho=0.6806, *p* < 0.001), suggesting that longer videos tended to provide more complete and reliable information. Major quality and correlation analyses are summarized in Table 3. Distributions of GQS and mDISCERN scores by uploader type are shown in Figures 4, 5, respectively. The correlation heatmap is shown in Figure 6. The cumulative distribution of comment counts is shown in Figure 7. In the exploratory ROC analysis, 41 videos had a final GQS of 4 or higher. Video duration discriminated these high-quality videos with an AUC of 0.886 (95% bootstrap CI, 0.834–0.934). A duration of 160 s maximized the Youden index, with a sensitivity of 95.1% and a specificity of 80.6%. In the multivariable sensitivity analysis adjusting for log-transformed duration and platform, non-medical uploader status was no longer significantly associated with final GQS (B = 0.192, 95% CI, −0.251 to 0.636, *p* = 0.395), whereas log-transformed duration remained positively associated with final GQS (B = 0.641, 95% CI, 0.530–0.752, *p* < 0.001; model R^2^ = 0.537). Detailed *post hoc* results are presented in Supplementary Table S2.

**Table 3:** Summary of major quality, correlation, and between-group analyses.

Indicator	Result
Mean GQS score of videos uploaded by medical professionals	2.72
Mean GQS score of videos uploaded by non-medical professionals	3.24
Between-group difference in GQS score	Mann–Whitney U = 1198.5, *p* = 0.033
Mean mDISCERN score of videos uploaded by medical professionals	2.41
Mean mDISCERN score of videos uploaded by non-medical professionals	2.52
Interrater agreement for GQS	ICC = 0.905
Between-group difference in mDISCERN score	Mann–Whitney U = 1490.5, *p* = 0.412
Interrater agreement for mDISCERN	ICC = 0.939
Video duration vs. likes	r = 0.053, *p* = 0.476
Video duration vs. shares	r = 0.145, *p* = 0.118
Video duration vs. collections	r = 0.251, *p* = 0.006
Video duration vs. GQS score	rho = 0.7434, *p* < 0.001
Video duration vs. mDISCERN score	rho = 0.6806, *p* < 0.001
Correlation between GQS and mDISCERN scores in videos uploaded by medical professionals	r = 0.8060, *p* < 0.001
Correlation between GQS and mDISCERN scores in videos uploaded by non-medical professionals	r = 0.7195, *p* < 0.001

### 3.4 Cross-platform comparisons

Kruskal–Wallis H tests showed significant differences across platforms in video duration (H = 36.508, *p* < 0.001), likes (H = 21.257, *p* < 0.001), comments (H = 44.904, *p* < 0.001), GQS scores (H = 18.104, *p* < 0.001), and mDISCERN scores (H = 16.419, *p* < 0.001). The chi-square test also showed significant differences in uploader type distribution across platforms (chi-square=31.155, *p* < 0.001). Exploratory *post-hoc* analyses indicated that TikTok differed significantly from Bilibili and YouTube in video duration, likes, comments, and GQS scores after Bonferroni correction; for mDISCERN, TikTok differed significantly from YouTube but not from Bilibili after correction. Pairwise differences between Bilibili and YouTube were not statistically significant after correction for these continuous variables. Cross-platform characteristics and inferential comparisons are presented in Tables 4, 5, respectively. Detailed pairwise comparisons are presented in Supplementary Table S1.

**Table 4:** Cross-platform comparison of dissemination characteristics, engagement, and video quality.

Platform	Videos, *n*	Medical uploaders, %	Duration, median (s)	Likes, median	Comments, median	Mean GQS	Mean mDISCERN
TikTok	93	100.0	66	239	21	2.45	2.10
Bilibili	24	62.5	445	57	3	3.29	2.60
YouTube	63	81.0	209	66	2	3.07	2.82

Video duration, likes, and comments are presented as medians. GQS and mDISCERN are presented as mean scores. Because some platforms did not display all engagement indicators, collections and shares were not included in the cross-platform comparison.

**Table 5:** Inferential statistical comparisons by uploader type and platform.

Comparison	Variable	Test statistic	*P* value
Medical vs. non-medical uploaders	Video duration	Mann–Whitney U = 1121.0	0.015
Medical vs. non-medical uploaders	Likes	Mann–Whitney U = 2308.5	0.004
Medical vs. non-medical uploaders	Collections	Mann–Whitney U = 519.0	0.740
Medical vs. non-medical uploaders	Shares	Mann–Whitney U = 580.0	0.339
Medical vs. non-medical uploaders	Comments	Mann–Whitney U = 2399.0	0.001
Medical vs. non-medical uploaders	GQS score	Mann–Whitney U = 1198.5	0.033
Medical vs. non-medical uploaders	mDISCERN score	Mann–Whitney U = 1490.5	0.412
Across platforms	Uploader type distribution	chi-square = 31.155	<0.001
Across platforms	Video duration	Kruskal–Wallis H = 36.508	<0.001
Across platforms	Likes	Kruskal–Wallis H = 21.257	<0.001
Across platforms	Comments	Kruskal–Wallis H = 44.904	<0.001
Across platforms	GQS score	Kruskal–Wallis H = 18.104	<0.001
Across platforms	mDISCERN score	Kruskal–Wallis H = 16.419	<0.001

## 4 Discussion

### 4.1 Principal findings

This cross-sectional content analysis systematically evaluated the content, quality, reliability, and dissemination characteristics of PD-related videos on TikTok, Bilibili, and YouTube. The findings show that although many videos had some educational value, overall information remained limited by uneven content coverage, inconsistent quality, and moderate-to-low reliability. These findings are consistent with previous studies of online videos on liver cancer, inflammatory bowel disease, gallstone disease, meniscal injury, and lymphedema, which reported incomplete information, variable quality, and limited evidence transparency (11–13, 16–18).

### 4.2 Uploader source, content coverage, and video quality

Medical professionals were the dominant creators of PD-related videos, suggesting that communication about this complex procedure is largely led by individuals or institutions with professional backgrounds. Previous short-video studies have similarly shown that medical content is often created by physicians or healthcare professionals, while quality and engagement can differ substantially across uploader types (11–13, 16). In the present study, videos uploaded by medical professionals received more likes and comments, indicating that users may be more inclined to engage with videos carrying professional identity cues when seeking information about complex surgical topics. However, engagement advantages did not necessarily translate into higher overall quality. Perceived credibility on social media is influenced not only by professional expertise but also by language style, trustworthiness, authenticity, and platform-specific cues (19–21). Although non-medical videos had higher GQS scores in the unadjusted comparison, they were also significantly longer, and video duration was strongly correlated with GQS. In the *post hoc* sensitivity analysis adjusting for video duration and platform, uploader type was no longer significantly associated with GQS, whereas duration remained a significant positive predictor. Therefore, the unadjusted GQS difference should not be interpreted as evidence that non-medical uploaders inherently produce higher-quality information. The clearer structure and public-oriented presentation sometimes observed among science popularization bloggers and non-profit medical institutions remain plausible explanations, but these possibilities require confirmation in larger and more balanced samples.

The content structure of PD-related videos was imbalanced. Videos focused primarily on surgical procedures or management, while disease background, indications, perioperative risks, postoperative complication recognition, and long-term outcomes were less frequently addressed. For a high-risk operation such as PD, patients need more than a visual explanation of how surgery is performed. They also need to understand when surgery is indicated, what problems should be monitored during recovery, and how complications can be recognized early (3–5, 8). Abnormal drainage appearance, persistent fever, worsening abdominal pain, recurrent jaundice, and feeding intolerance may signal clinically important complications, yet information directly relevant to patient safety was insufficient in many videos. Research on online cancer risk communication has similarly shown that unexplained medical terminology, insufficient credibility cues, and unclear risk presentation can hinder public understanding of complex health information (22).

### 4.3 Video duration, engagement, and two-way communication

Video duration was positively correlated with both GQS and mDISCERN, suggesting that longer videos may allow more complete explanations and more reliable information. Similar associations between video duration and information quality or reliability have been reported in short-video studies of gallstone disease and inflammatory bowel disease (12, 13). In the exploratory ROC analysis, a duration of 160 s was the data-derived cut point that best distinguished videos with a final GQS of 4 or higher in this sample. This finding suggests that approximately 2.5–3 min may provide sufficient time to present a more structured explanation of a complex surgical topic. However, the threshold was derived *post hoc* from the current sample, was not externally validated, and should not be treated as a universal minimum or causal recommendation. Content accuracy, organization, transparency, and audience suitability remain more important than duration alone. Moreover, duration was not significantly associated with likes or shares and was only weakly correlated with collections. Thus, more complete videos may not receive immediate dissemination advantages, although users may perceive them as useful reference materials. Engagement with online health content is shaped by multiple factors, including accuracy, comprehensibility, visual presentation, completeness, credibility, and interactivity (23, 24). Medical professionals may provide more accurate information but may also use technical anatomical terms or specialist language that increases the cognitive burden for lay viewers. Non-medical creators may present information from the patient perspective using more accessible narratives, potentially improving comprehension and audience acceptance (20, 21, 25).

Comment patterns also suggest that many videos did not generate effective two-way communication. For a complex and preference-sensitive surgical topic such as PD, one-way information delivery may be insufficient to meet the needs of patients and families. Prior research has shown that patients often seek online information when clinical consultations do not fully address their informational needs, while clinician recommendation of reliable digital resources remains limited (6, 7). More structured question-and-answer mechanisms, creator responses in comment sections, or links to authoritative resources may improve the efficiency and accuracy of patient information seeking.

### 4.4 Platform differences

Platform differences were also evident. TikTok videos had the highest engagement and the shortest duration but lower quality and reliability scores. Bilibili videos were longer and had relatively limited engagement but showed a higher mean GQS in this sample. YouTube showed a more balanced profile between dissemination and quality, with a higher mean mDISCERN in this sample. Prior research suggests that social media platforms differ in information presentation, user engagement, and misinformation dynamics, and short-video platforms such as TikTok are increasingly used by younger populations as health information sources (6, 9, 10, 26). These findings indicate that platform-level dissemination efficiency does not necessarily correspond to medical information quality. Nevertheless, the observed platform differences may partly reflect platform-specific ranking systems and the composition of highly ranked search results rather than intrinsic differences between all videos available on each platform. For complex surgical topics, platforms that allow longer content and more systematic explanations may be more suitable for reliable patient education (12, 13, 18).

### 4.5 Practical implications

Several practical implications follow from these findings. Clinicians and professional creators should avoid limiting PD-related videos to operative scenes or procedural demonstrations. Instead, content should be structured around a patient education framework that includes Definition, Symptoms/Indications, Management, and Outcomes. Graphic surgical scenes should be reduced when they do not improve understanding, while three-dimensional animation, diagrams, flowcharts, and short animations can be used to explain complex anatomy and surgical steps. Educational videos and animations have been shown to improve patient understanding of difficult medical terminology, health information recall, treatment adherence, and perioperative psychological outcomes (27–30). Patients and families should recognize that short videos are fragmented information sources and should not rely on them alone for major medical decisions. They should prioritize systematic educational resources from official medical institutions or authoritative specialist teams and use short videos as supplements to clinical consultations (6, 7, 31). Platform managers should improve health content verification, risk labeling, and recommendation mechanisms to increase the visibility and credibility of high-quality content. Medical professionals also need digital health communication skills and awareness of e-professionalism to participate effectively in public-facing health communication (19, 32, 33).

### 4.6 Limitations

This study has several limitations. First, mDISCERN and GQS scoring involved manual assessment, and although interrater agreement was excellent, subjective judgment may still have introduced bias. Second, short-video platforms are highly dynamic. Video rankings, engagement indicators, and recommendation algorithms can change over time; therefore, the findings reflect platform content during the search period (10, 11). Although newly registered accounts reduced the influence of prior browsing history, they could not remove default algorithmic ranking. The first 100 eligible TikTok and YouTube videos consequently represented highly ranked results under specific search conditions rather than random samples, and ranking may have been affected by popularity, recency, geographic location, language, and proprietary algorithms. Third, engagement analysis was based on quantitative indicators such as likes, comments, collections, and shares and did not include qualitative analysis of comment content. Therefore, viewers’ detailed information needs and creators’ responsiveness could not be fully evaluated. Fourth, the study focused on patient education and public health communication and did not assess the value of these videos for medical students or professional continuing education.

Additional limitations relate to sample composition and variables not prospectively collected. The final platform samples were unequal (93 TikTok, 24 Bilibili, and 63 YouTube videos), particularly because relatively few eligible Bilibili videos were available. This imbalance may reduce statistical power and the stability and generalizability of cross-platform comparisons; such comparisons should therefore be interpreted as exploratory. Uploader type, platform, and video duration were also not fully balanced. Consistent with the *post hoc* adjusted analysis, the higher unadjusted GQS among non-medical videos should not be interpreted as an independent effect of uploader identity. Sponsorship status and account follower counts were not prespecified variables. Re-review of the archived records identified no videos with an explicit sponsorship, paid-partnership, or advertising disclosure. A video posted by a hospital official account and promoting the institution itself was retained in the hospital-account category and was not classified as sponsored in the absence of an external sponsorship disclosure. Undisclosed commercial relationships could not be reliably inferred. Follower counts were not recorded at the original data-collection time point, and retrospective collection would create a temporal mismatch because follower counts are dynamic. Finally, the search terms were centered primarily on PD and the Whipple procedure. This strategy may have preferentially retrieved procedural content while missing videos framed around symptoms, postoperative care, complications, recovery, patient education, or broader pancreatic cancer management. Future studies should prospectively record explicit sponsorship disclosures and time-matched follower counts, use broader patient-oriented search terms, and validate the exploratory 160-s duration threshold in independent samples.

## 5 Conclusion

This study evaluated the content, quality, reliability, and dissemination characteristics of 180 PD-related videos on TikTok, Bilibili, and YouTube. Videos uploaded by both medical and non-medical professionals had some educational value, but overall content remained incomplete, quality was inconsistent, and reliability was limited. Current videos primarily emphasized surgical procedures and management while underrepresenting disease definitions, indications, postoperative outcomes, and prognosis. Future PD-related short videos should improve accuracy, completeness, comprehensibility, and transparency. Platforms should strengthen health information governance and support users in identifying trustworthy sources, thereby helping short-video platforms become more reliable channels for patient education and public health communication.

## Data Availability

The raw data supporting the conclusions of this article will be made available by the authors, without undue reservation.

## Supplementary material

Supplementary Material Supplementary Tables S1 and S2 presenting detailed post hoc platform comparisons and supplementary regression analyses.

## References

[1] Klein AP. Pancreatic cancer epidemiology: understanding the role of lifestyle and inherited risk factors. Nat Rev Gastroenterol Hepatol. (2021) 18:493–502. 10.1038/s41575-021-00457-x 34002083 PMC9265847

[2] Donahue TR Reber HA. Surgical management of pancreatic cancer-pancreaticoduodenectomy. Semin Oncol. (2015) 42:98–109. 10.1053/j.seminoncol.2014.12.009 25726055

[3] Yoon Y-S Lee W Kang CM Hong T Shin SH Lee JW. Laparoscopic versus open pancreatoduodenectomy for periampullary tumors: a randomized clinical trial. Int J Surg. (2024) 110:7011–9. 10.1097/JS9.0000000000002035 39172725 PMC11573048

[4] Ong BDC Goh WJWH Tan MMT Loke S Kan JRY Yeo JKW. Does minimally invasive pylorus-preserving pancreaticoduodenectomy (MI-PPPD) triumph over open pylorus-preserving pancreaticoduodenectomy (O-PPPD)? A systematic review and meta-analysis. Int J Surg. (2026) 112:10603–11. 10.1097/JS9.0000000000004734 41549828 PMC13105632

[5] Robertson RH Russell K Jordan V Pandanaboyana S Wu D Windsor J. Postoperative nutritional support after pancreaticoduodenectomy in adults. Cochrane Database Syst Rev. (2025) 3:CD014792. 10.1002/14651858.CD014792.pub2 40084692 PMC11907764

[6] Stifjell K Sandanger TM Wien C. Exploring online health information-seeking behavior among young adults: scoping review. J Med Internet Res. (2025) 27:e70379. 10.2196/70379 40925001 PMC12457860

[7] Erbas ME Ziehfreund S Wecker H Biedermann T Zink A. Digital media usage behavior and its impact on the physician-patient relationship: cross-sectional study among individuals affected by psoriasis in Germany. J Med Internet Res. (2024) 26:e57823. 10.2196/57823 39110972 PMC11339573

[8] Ten Haaft BHEA Janssen BV Barsom EZ Hehenkamp WJK van Berge Henegouwen MI Busch OR. Online video versus face-to-face preoperative consultation for major abdominal surgery (VIDEOGO): a multicentre, open-label, randomised, controlled, non-inferiority trial. Lancet Digit Health. (2025) 7:100867. 10.1016/j.landig.2025.02.007 40523832

[9] Yeung AWK Tosevska A Klager E Eibensteiner F Tsagkaris C Parvanov ED. Medical and health-related misinformation on social media: bibliometric study of the scientific literature. J Med Internet Res. (2022) 24:e28152. 10.2196/28152 34951864 PMC8793917

[10] Suarez-Lledo V Alvarez-Galvez J. Prevalence of health misinformation on social media: systematic review. J Med Internet Res. (2021) 23:e17187. 10.2196/17187 33470931 PMC7857950

[11] Zheng S Tong X Wan D Hu C Hu Q Ke Q. Quality and reliability of liver cancer-related short Chinese videos on TikTok and Bilibili: cross-sectional content analysis study. J Med Internet Res. (2023) 25:e47210. 10.2196/47210 37405825 PMC10357314

[12] He Z Wang Z Song Y Liu Y Kang L Fang X. The reliability and quality of short videos as a source of dietary guidance for inflammatory bowel disease: cross-sectional study. J Med Internet Res. (2023) 25:e41518. 10.2196/41518 36757757 PMC9951074

[13] Sun F Zheng S Wu J. Quality of information in gallstone disease videos on TikTok: cross-sectional study. J Med Internet Res. (2023) 25:e39162. 10.2196/39162 36753307 PMC9947761

[14] Lee N Oh SW Cho B Myung SK Hwang SS Yoon GH. A health information quality assessment tool for Korean online newspaper articles: development study. J Med Internet Res. (2021) 23:e24436. 10.2196/24436 34326038 PMC8367132

[15] Charnock D Shepperd S Needham G Gann R. DISCERN: an instrument for judging the quality of written consumer health information on treatment choices. J Epidemiol Community Health. (1999) 53:105–11. 10.1136/jech.53.2.105 10396471 PMC1756830

[16] Kunze KN Krivicich LM Verma NN Chahla J. Quality of online video resources concerning patient education for the meniscus: a YouTube-based quality-control study. Arthroscopy. (2020) 36:233–8. 10.1016/j.arthro.2019.07.033 31864582

[17] Zhou X Ma G Su X Li X Wang W Xia L. The reliability and quality of short videos as health information of guidance for lymphedema: a cross-sectional study. Front Public Health. (2024) 12:1472583. 10.3389/fpubh.2024.1472583 39830188 PMC11739071

[18] Perry NM Kelly JJ Levy BA. Editorial commentary: surgical videos on YouTube are not peer reviewed and have low educational value. Arthroscopy. (2024) 40:2244–5. 10.1016/j.arthro.2024.03.006 38467169

[19] Vukušić Rukavina T Viskić J Machala Poplašen L Relić D Marelić M Jokic D. Dangers and benefits of social media on e-professionalism of health care professionals: scoping review. J Med Internet Res. (2021) 23:e25770. 10.2196/25770 PMC8663533 34662284

[20] Quinn B Nichols L Frazee J Payton M Linger RMA. Dissemination of information on selective serotonin reuptake inhibitors on TikTok: analytical mixed methods study of creator types, content tone, and user engagement. JMIR Ment Health. (2025) 12:e77383. 10.2196/77383 41027037 PMC12483472

[21] Jenkins EL Ilicic J Barklamb AM McCaffrey TA. Assessing the credibility and authenticity of social media content for applications in health communication: scoping review. J Med Internet Res. (2020) 22:e17296. 10.2196/17296 32706675 PMC7413282

[22] Waters EA Foust JL Scherer LD McQueen A Taber JM. Adherence of internet-based cancer risk assessment tools to best practices in risk communication: content analysis. J Med Internet Res. (2021) 23:e23318. 10.2196/23318 33492238 PMC7870349

[23] Oktay LA Abuelgasim E Abdelwahed A Houbby N Lampridou S Normahani P. Factors affecting engagement in web-based health care patient information: narrative review of the literature. J Med Internet Res. (2021) 23:e19896. 10.2196/19896 34554104 PMC8498891

[24] Rus HM Cameron LD. Health communication in social media: message features predicting user engagement on diabetes-related Facebook pages. Ann Behav Med. (2016) 50:678–89. 10.1007/s12160-016-9793-9 27059761

[25] Feng B Malloch YZ Kravitz RL Verba S Iosif A-M Slavik G. Assessing the effectiveness of a narrative-based patient education video for promoting opioid tapering. Patient Educ Couns. (2021) 104:329–36. 10.1016/j.pec.2020.08.019 32900605 PMC7855718

[26] Grabb D. Leaning into the digital age: the role of TikTok and other technologies in providing mental health information. Br Med J. (2023) 382:p1754. 10.1136/bmj.p1754 37524394

[27] Pentz RD Lohani M Hayban M Switchenko JM Dixon MD DeFeo RJ. Videos improve patient understanding of misunderstood chemotherapy terminology. Cancer. (2019) 125:4011–8. 10.1002/cncr.32421 31418849 PMC6986301

[28] Sibold HC Thomson MC Hianik R Abernethy ER Campbell GP Sumrall B. Videos improve patient understanding of chemotherapy terminology in a rural setting. Cancer. (2021) 127:4015–21. 10.1002/cncr.33810 34289098 PMC8516682

[29] Shi X Wang Y Wang Y Wang J Peng C Cheng S. The effectiveness of digital animation-based multistage education for patients with atrial fibrillation catheter ablation: randomized clinical trial. J Med Internet Res. (2025) 27:e65685. 10.2196/65685 40067344 PMC11937711

[30] Hansen S Jensen TS Schmidt AM Strøm J Vistisen P Høybye MT. The effectiveness of video animations as a tool to improve health information recall for patients: systematic review. J Med Internet Res. (2024) 26:e58306. 10.2196/58306 39753224 PMC11730234

[31] Song S Zhang Y Yu B. Interventions to support consumer evaluation of online health information credibility: a scoping review. Int J Med Inform. (2021) 145:104321. 10.1016/j.ijmedinf.2020.104321 33202372

[32] Cai X Li W Shi W Cai Y Zhou J. Behavioral dynamics of AI trust and health care delays among adults: integrated cross-sectional survey and agent-based modeling study. J Med Internet Res. (2026) 28:e82170. 10.2196/82170 41632964 PMC12914233

[33] Car J Ong QC Erlikh Fox T Leightley D Kemp SJ Švab I. The digital health competencies in medical education framework: an international consensus statement based on a Delphi study. JAMA Netw Open. (2025) 8:e2453131. 10.1001/jamanetworkopen.2024.53131 39888625

