# Supplementary material for: Quality and reliability of pancreaticoduodenectomy-related videos on TikTok, Bilibili, and YouTube: a cross-sectional content analysis
Source: Front Surg. 2026 Sep 9;13:1898401. doi: 10.3389/fsurg.2026.1898401 (PMC13597764; doi:10.3389/fsurg.2026.1898401)
Supplement: Supplementary file 1 [file Table1.docx]

**Supplementary Table S1.** Exploratory pairwise platform comparisons after significant overall platform tests

| **Variable** | **Pairwise comparison** | **Test statistic** | **Adjusted p value** |
| --- | --- | --- | --- |
| Video duration | TikTok vs Bilibili | Dunn z=−5.140 | <0.001 |
| Video duration | TikTok vs YouTube | Dunn z=−4.521 | <0.001 |
| Video duration | Bilibili vs YouTube | Dunn z=1.831 | 0.202 |
| Likes | TikTok vs Bilibili | Dunn z=2.908 | 0.011 |
| Likes | TikTok vs YouTube | Dunn z=4.264 | <0.001 |
| Likes | Bilibili vs YouTube | Dunn z=0.125 | 1.000 |
| Comments | TikTok vs Bilibili | Dunn z=3.211 | 0.004 |
| Comments | TikTok vs YouTube | Dunn z=6.557 | <0.001 |
| Comments | Bilibili vs YouTube | Dunn z=1.395 | 0.489 |
| GQS score | TikTok vs Bilibili | Dunn z=−3.341 | 0.003 |
| GQS score | TikTok vs YouTube | Dunn z=−3.484 | 0.001 |
| GQS score | Bilibili vs YouTube | Dunn z=0.819 | 1.000 |
| mDISCERN score | TikTok vs Bilibili | Dunn z=−1.683 | 0.277 |
| mDISCERN score | TikTok vs YouTube | Dunn z=−4.014 | <0.001 |
| mDISCERN score | Bilibili vs YouTube | Dunn z=−1.124 | 0.782 |
| Uploader type distribution | TikTok vs Bilibili | Fisher exact test | <0.001 |
| Uploader type distribution | TikTok vs YouTube | Fisher exact test | <0.001 |
| Uploader type distribution | Bilibili vs YouTube | Fisher exact test | 0.282 |

Note: Dunn tests with Bonferroni correction were used for pairwise comparisons of continuous variables after Kruskal–Wallis tests. Fisher exact tests with Bonferroni correction were used for exploratory pairwise comparisons of uploader type distribution. Adjusted p values are shown.

**Supplementary Table S2.** Exploratory analyses of video duration and final Global Quality Score

**A. Receiver operating characteristic analysis**

| **Indicator** | **Result** |
| --- | --- |
| Analysis population | All 180 included videos |
| High-quality outcome | Final GQS ≥4 (good-to-excellent quality) |
| High-quality videos, n | 41 |
| Other videos, n | 139 |
| Area under the curve | 0.886 |
| 95% bootstrap confidence interval | 0.834–0.934 |
| Youden-index duration threshold | 160 s |
| Sensitivity at threshold | 95.1% |
| Specificity at threshold | 80.6% |

**B. Multivariable linear regression sensitivity analysis**

| **Predictor** | **B** | **95% CI** | **p value** |
| --- | --- | --- | --- |
| Non-medical uploader (vs medical) | 0.192 | −0.251 to 0.636 | 0.395 |
| ln[duration (s) + 1] | 0.641 | 0.530 to 0.752 | <0.001 |
| Bilibili (vs TikTok) | −0.284 | −0.749 to 0.182 | 0.232 |
| YouTube (vs TikTok) | −0.061 | −0.314 to 0.193 | 0.640 |

Model R²=0.537. Final GQS was the dependent variable. Heteroscedasticity-robust (HC3) standard errors were used.

Note: These analyses were post hoc and exploratory. The ROC confidence interval was estimated using 5,000 bootstrap resamples. The duration threshold is sample-dependent and should not be interpreted as a universal minimum duration for high-quality health videos. GQS, Global Quality Score; ROC, receiver operating characteristic; CI, confidence interval.
